# Beyond complication rates: Nationwide neurosurgical compensation claims in a No-fault system

**DOI:** 10.1016/j.bas.2026.107470

**Published:** 2026-10-07

**Authors:** F.M.C. Lioi, N. Sunthara, M.K.H. Wiedmann

**Affiliations:** a Department of Neurosurgery - Policlinico "Tor Vergata", University of Rome "Tor Vergata", Rome, Italy; b Department of Neurosurgery, Oslo University Hospital, Oslo, Norway; c Institute of Clinical Medicine, University of Oslo, Oslo, Norway

**Keywords:** Adverse events, Compensable harm, Compensation decisions, Malpractice, Neurosurgery, No-fault compensation, Patient safety

## Abstract

**Background and objectives:**

Medico-legal claims are often used as proxies for malpractice burden and health care quality. However, they reflect not only underlying clinical events but also the structure of the compensation system through which they are adjudicated. This is particularly relevant in neurosurgery, where high baseline risk and uncertainty in outcome attribution may influence both the propensity to file claims and their subsequent resolution. The objective of this study was to characterize compensation outcomes, clinical domains, decision grounds, and failure-process patterns in a nationwide no-fault neurosurgical dataset.

**Methods:**

We retrospectively analyzed 620 neurosurgery-related compensation decisions issued in Norway between 2015 and 2024, examining clinical domains, decision grounds, and failure-process patterns.

**Results:**

Compensation was granted in 93 of 620 decisions (15.0%). Nontraumatic spinal disorders represented the largest domain (225/620, 36.3%) but had a low grant rate (23/225, 10.2%), whereas grant rates were higher in cerebrospinal fluid and shunt disorders (17/43, 39.5%) and trauma (9/29, 31.0%). Within nontraumatic spinal disorders, cervical degenerative disease showed particularly low compensability (8/149, 5.4%). Among granted decisions, the leading failure-process domains were preoperative or diagnostic (35/93, 37.6%), intraoperative or procedural (22/93, 23.7%), and perioperative or postoperative management (13/93, 14.0%).

**Conclusion:**

In this nationwide neurosurgical series from a no-fault compensation system, compensation was granted in 15.0% of decisions and primarily reflected avoidable failures along the care pathway rather than adverse outcomes alone. Compensation decision data should therefore not be interpreted as complication rates, but as system-filtered signals of compensable and avoidable harm shaped by adjudicatory framework and system design.

## Highlights

• Nationwide study of 620 neurosurgical decisions in a no-fault system.
• Compensation was granted in only 15.0% of final decisions.
• CSF and shunt disorders had the highest compensation rate (39.5%).
• Diagnostic and perioperative failures predominated among granted decisions.
• Compensation decisions signal avoidable harm, not surgical complication rates.

## Abbreviations, nomenclature and symbols

CI: confidence interval
Cramér V: effect size measure for categorical association
CSF: cerebrospinal fluid
ICD-10: International Classification of Diseases, 10th Revision
NOK: Norwegian krone
NOMESCO: Nordic Medico-Statistical Committee
NPE: Norwegian System of Patient Injury Compensation (Norsk pasientskadeerstatning)
OR: odds ratio
STROBE: Strengthening the Reporting of Observational Studies in Epidemiology
χ^2^(: chi-square

## 1 Introduction

Medico-legal claim data are often used as indicators of malpractice burden, patient safety, and health care quality (Mukherjee et al., 2014; Steele et al., 2015; Machin et al., 2018; Dronkers et al., 2025). However, they do not directly measure adverse-event or complication rates. Rather, they reflect which events are submitted, reviewed, and judged compensable within a specific compensation pathway. This distinction is especially important in neurosurgery, where high baseline risk, disease-related morbidity, and uncertainty in outcome attribution may obscure the boundary between unavoidable outcomes and avoidable failures in care. Centralized no-fault compensation systems provide a distinctive opportunity to study these patterns because claims are assessed within a single administrative framework rather than through fragmented litigation pathways (Pukk-Härenstam et al., 2008; Öhrn et al., 2011; Tilma et al., 2015, 2016; Smeby et al., 2015). Norway is well suited for such analysis because neurosurgery-related injury claims are reviewed through the Norwegian System of Patient Injury Compensation (Norsk pasientskadeerstatning [NPE]), a national no-fault compensation scheme (Ministry of Health and Care Service, 2007; What are the criteria to, What does the compensation cover). We analyzed a nationwide 10-year series of neurosurgery-related compensation decisions issued in Norway, aiming to describe compensation outcomes, clinical domains, administrative decision grounds, and failure-process patterns, and to clarify what no-fault compensation data represent as medico-legal and patient-safety signals.

## 2 Methods

### 2.1 Study design, reporting, and data source

We conducted a retrospective observational study of neurosurgery-related compensation decisions issued in Norway between 2015 and 2024. Reporting followed the STROBE statement using the checklist for cross-sectional studies (von et al., 2008). Study material was obtained from NPE after formal application by the senior author. NPE is the national administrative no-fault compensation scheme for patient injuries in Norway; compensation may be granted when injury is causally linked to failure in examination, diagnosis, treatment, or follow-up, or under a statutory exception clause for serious and unexpected injury. NPE decisions are based on medical records, statements from the claimant and treatment provider, and assessments from internal or external medical experts when relevant. Medical experts provide a professional assessment of the clinical aspects of diagnosis, treatment, and follow-up, while the final administrative determination regarding entitlement to compensation is made by NPE. NPE decisions may be appealed; accordingly, the term "final compensation decision" in this study refers to the administrative decision contained in the dataset supplied by NPE and does not imply that no subsequent appeal or legal remedy was available. Following administrative approval, NPE provided deidentified records of these decisions. Because the dataset contained no directly identifiable patient information, ethics committee review and informed consent were not required under applicable regulations. After verification of dataset completeness, the final analytic cohort comprised 620 compensation decisions. NPE also provided a separate aggregated extract comprising 503 decisions with annual counts and compensation amounts. Because this extract could not be linked to the main cohort at the individual-decision level, temporal and economic analyses were performed separately.

### 2.2 Variables and classifications

The primary outcome was the final compensation decision, classified as granted or denied. Available variables included sex, age at injury in decade-based categories, primary and secondary ICD-10 diagnosis codes, NOMESCO procedure codes, and a brief deidentified narrative summary (ICD-10 Version, Nomesco, 2011). Main neurosurgical domains were assigned primarily according to the primary ICD-10 diagnosis code. Records with ambiguous, overlapping, or competing classifications were independently reviewed by 2 authors and resolved by consensus. Mutually exclusive domains were nontraumatic spinal disorders, tumors, cerebrovascular disorders, cerebrospinal fluid and shunt disorders, trauma, functional disorders, and miscellaneous conditions. Within nontraumatic spinal disorders, decisions were subdivided into cervical degenerative disease (M50*), other degenerative spinal disorders, and other nondegenerative spinal diagnoses. Other degenerative spinal disorders comprised remaining degenerative M* diagnoses after exclusion of clearly nondegenerative conditions, including scoliosis, spinal infection, nonspecific back pain, and peripheral neuralgia. For the specific comparison of cervical degenerative disease reported in the Results, the latter two categories were pooled as "remaining spinal diagnoses." Cerebrovascular decisions were assigned using the primary diagnosis field and a predefined vascular ICD-10 framework (I60-I65, I67.1, I67.5, Q28*, I77.0, and G45*), with manual review of borderline or overlapping records to ensure a single final domain assignment. Claim-level event categories were derived from coded NPE variables and included process-of-care problems, neurologic injury, pain, diagnostic delay or error, local complications, system-, medication-, or patient-information-related events, and other or unspecified categories. For the purposes of this analysis, process-of-care problems referred to the administrative event category describing problems in the organization or delivery of care rather than a specific resulting clinical outcome such as neurologic injury or pain. These event categories were mutually exclusive administrative coding categories and should not be interpreted as mutually exclusive causal mechanisms in a clinical sense. Failure-process domains were derived from coded variables and narrative review. Emergency or nonemergency status was derived from the narrative summary when sufficient information was available.

### 2.3 Decision grounds and failure-process coding

Final decision grounds were categorized according to the administrative rationale recorded in the NPE files. Denied decisions were grouped as no failure in diagnosis, no causal link, no failure in treatment, or other grounds. Granted decisions were categorized according to the principal basis for compensation, including failure in diagnosis, failure in treatment, statutory exception, infection, patient-communication failures, device- or equipment-related errors, or other grounds. In the NPE framework, treatment failure is an administrative category referring broadly to injury related to an error or omission in treatment, examination, diagnosis, or follow-up and should not be interpreted as synonymous with technical intraoperative error. Where available, granular cause-of-failure variables were reviewed and grouped into clinically meaningful failure-process domains: preoperative or diagnostic, intraoperative or procedural, perioperative or postoperative management, follow-up or surveillance, system-level or communication-related, competence-related, and other or insufficiently specified. Granular NPE cause-of-failure variables and the broader clinically oriented failure-process classification therefore represent different levels of classification. The latter could also be assigned from narrative review when sufficiently detailed information was available. Because these variables were incompletely available, failure-process patterns were analyzed descriptively with explicit denominators.

### 2.4 Statistical analysis

The unit of analysis was the final compensation decision. Categorical variables are reported as counts and percentages. Associations with compensation outcome were assessed using the χ^2^ test or Fisher exact test, as appropriate. When overall associations were significant, exploratory post hoc comparisons were performed using Fisher exact tests with Holm correction for multiple testing. ORs with 95% CIs are reported for selected binary comparisons. Missing data were handled by available-case analysis, with no imputation. All tests were 2-sided, and P < .05 was considered statistically significant. Statistical analyses were performed using JASP, version 0.97.0 (JASP Team, University of Amsterdam, Amsterdam, the Netherlands), and R, version 4.6.0 (R Foundation for Statistical Computing, Vienna, Austria).

## 3 Results

### 3.1 Cohort characteristics and clinical domains

The final analytic cohort comprised 620 compensation decisions, including 93 granted decisions (15.0%) and 527 denied decisions (85.0%) (Table 1 and Fig. 1). Of these decisions, 311 involved male patients (50.2%) and 309 involved female patients (49.8%); 321 decisions (51.8%) involved patients aged 40 to 59 years. Compensation outcome was not significantly associated with sex (P = .52) or age group (P = .85). Nontraumatic spinal disorders constituted the largest domain (225/620, 36.3%), followed by tumors (140/620, 22.6%), miscellaneous conditions (77/620, 12.4%), cerebrovascular disorders (63/620, 10.2%), cerebrospinal fluid and shunt disorders and functional disorders (43/620, 6.9% each), and trauma (29/620, 4.7%) (Fig. 1). Compensation rates differed significantly across domains (P < .001; Cramér V = 0.23), ranging from 10.2% in nontraumatic spinal disorders to 39.5% in cerebrospinal fluid and shunt disorders. In exploratory pairwise comparisons with Holm correction, the cerebrospinal fluid and shunt domain had higher grant rates than nontraumatic spinal disorders (OR, 5.74; 95% CI, 2.72-12.13; adjusted P = .00021), tumors (OR, 4.73; 95% CI, 2.14-10.47; adjusted P = .00375), and cerebrovascular disorders (OR, 4.50; 95% CI, 1.72-11.75; adjusted P = .0413).

**Table 1:** Cohort characteristics and claim-level event categories according to compensation outcome.

**Characteristic or claim-level event category**	All decisions (n = 620), No. (%)	Granted decisions (n = 93), No. (%)	Denied decisions (n = 527), No. (%)	P value
**Total decisions**	620 (100)	93 (100)	527 (100)	—
**Sex**				0.52
Men	311 (50.2)	50 (53.8)	261 (49.5)	
Women	309 (49.8)	43 (46.2)	266 (50.5)	
**Age group, y**				0.85
0-9	25 (4.0)	5 (5.4)	20 (3.8)	
10-19	21 (3.4)	3 (3.2)	18 (3.4)	
20-29	43 (6.9)	7 (7.5)	36 (6.8)	
30-39	84 (13.5)	10 (10.8)	74 (14.0)	
40-49	164 (26.5)	20 (21.5)	144 (27.3)	
50-59	157 (25.3)	27 (29.0)	130 (24.7)	
60-69	90 (14.5)	14 (15.1)	76 (14.4)	
≥70	36 (5.8)	7 (7.5)	29 (5.5)	
**Claim-level event category**				<0.001
Process-of-care problems	310 (50.0)	26 (28.0)	284 (53.9)	
Neurologic injury	89 (14.4)	4 (4.3)	85 (16.1)	
Pain	51 (8.2)	3 (3.2)	48 (9.1)	
Diagnostic delay or error	30 (4.8)	8 (8.6)	22 (4.2)	
Local complications	21 (3.4)	7 (7.5)	14 (2.7)	
System-, medication-, or patient-information–related events	11 (1.8)	7 (7.5)	4 (0.8)	
Other or unspecified event category	108 (17.4)	38 (40.9)	70 (13.3)	

**Note.** Claim-level event categories are mutually exclusive categories derived from administrative injury-type coding and describe the main injury, event, or process problem addressed in the decision. They should not be interpreted as mutually exclusive causal mechanisms in a clinical sense; for example, neurological injury may result from a process-of-care problem. “Other or unspecified” is shown last because it represents a residual administrative category, irrespective of frequency. In denied decisions, these categories should not be interpreted as confirmed compensable injuries.

**Fig. 1:**
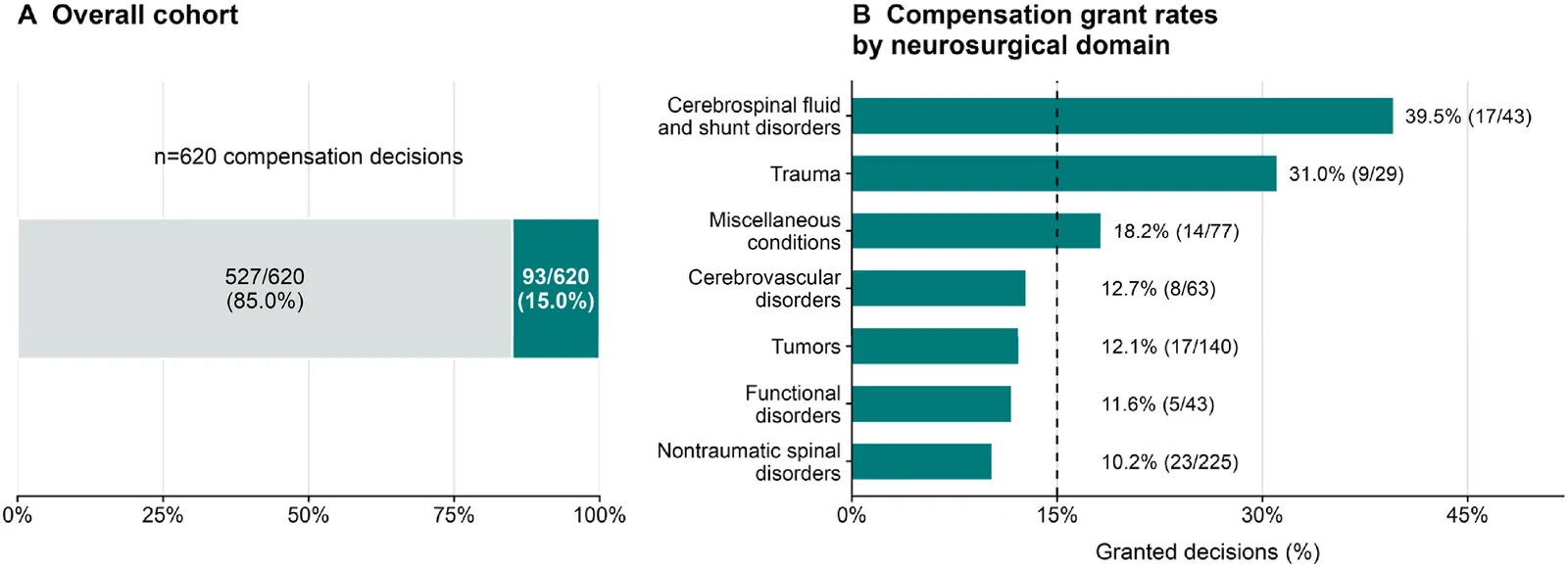
The figure depicts the overall cohort and compensation outcomes and variation in grant rates across neurosurgical domains.

### 3.2 Compensation patterns by clinical domain

Selected domain-level and within-domain subgroup patterns are shown in Fig. 2. Within nontraumatic spinal disorders, cervical degenerative disease accounted for 149 of 225 decisions (66.2%) and had a lower grant rate than the remaining spinal diagnoses, which pooled other degenerative and nondegenerative spinal diagnoses for this comparison (8/149 [5.4%] vs 15/76 [19.7%]; OR, 0.23; 95% CI, 0.09-0.57; Fisher exact P = .002). Within tumors, grant rates were 15.9% (7/44) for meningiomas, 7.1% (2/28) for glioma-coded tumors, 5.3% (1/19) for benign pituitary and sellar lesions including craniopharyngeal duct tumors, and 14.3% (7/49) for other tumors. Grant rates were relatively low in cerebrovascular disorders (8/63, 12.7%) and functional disorders (5/43, 11.6%). Among selected miscellaneous subgroups, grant rates were 21.4% (3/14) for cystic or structural nontumor lesions, 16.7% (1/6) for infection or abscess, and 18.8% (3/16) for congenital nonvascular anomalies. The cerebrospinal fluid and shunt domain had the highest overall compensation rate (17/43, 39.5%); within this domain, hydrocephalus and cerebrospinal fluid circulation disorders accounted for most decisions (34/43, 79.1%) and had a grant rate of 41.2% (14/34). Of the 43 CSF/shunt-related decisions, 11 (25.6%) involved patients aged 0-19 years and 32 (74.4%) involved patients aged 20 years or older. Among the 17 granted decisions in this domain, 3 (17.6%) involved patients aged 0-19 years. Age was available only in decade-based categories, so an 18-year cutoff could not be applied. In trauma, spinal trauma had a numerically higher grant rate than cranial trauma (6/15 [40.0%] vs 3/14 [21.4%]).

**Fig. 2:**
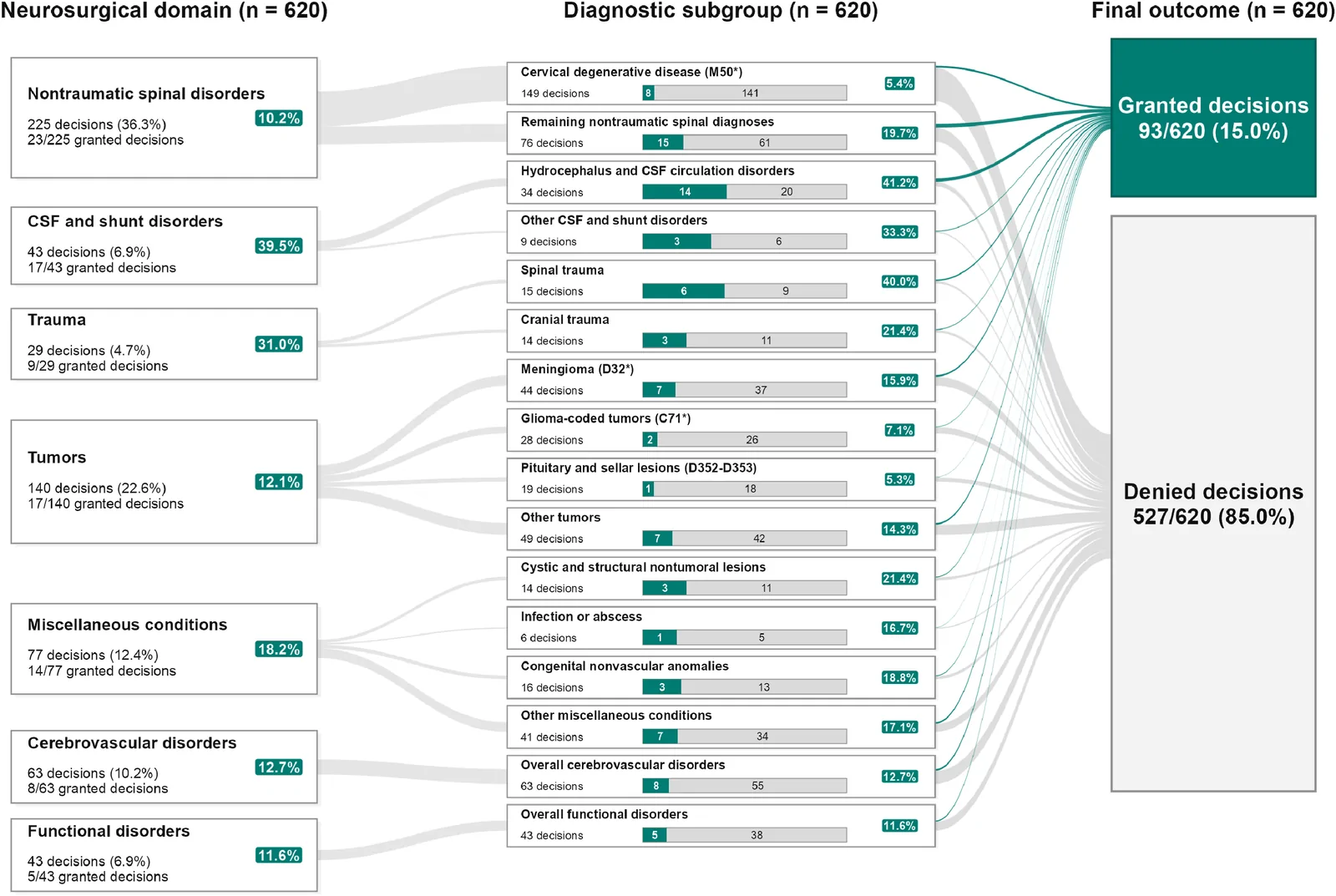
**Domain, selected subgroup, and outcome distribution.** Alluvial diagram of 620 neurosurgery-related compensation decisions. Ribbon width is proportional to decision volume; green indicates granted decisions and gray denied decisions. Grant-rate badges show the percentage of granted decisions within each domain or subgroup. Selected ICD-10 anchors are shown where appropriate.

### 3.3 Claim-level event categories and decision grounds

Claim-level event categories are summarized in Table 1. Process-of-care problems were the most frequent category (310/620, 50.0%), followed numerically by the residual other or unspecified category (108/620, 17.4%). Among the specified clinical event categories, neurologic injury was the most frequent (89/620, 14.4%), followed by pain (51/620, 8.2%). The distribution of claim-level event categories differed significantly by compensation outcome (P < .001). In exploratory category-level comparisons with Holm correction, neurologic injury codes were underrepresented among granted decisions (4/93 [4.3%] vs 85/527 [16.1%]; OR, 0.23; 95% CI, 0.08-0.65; adjusted P = .006), whereas system-, medication-, or patient-information-related events were overrepresented (7/93 [7.5%] vs 4/527 [0.8%]; OR, 10.64; 95% CI, 3.05-37.13; adjusted P = .001). Local complications showed a directional but nonsignificant difference after correction (7/93 [7.5%] vs 14/527 [2.7%]; OR, 2.98; 95% CI, 1.17-7.60; adjusted P = .053), whereas diagnostic delay or error did not differ significantly after correction (8/93 [8.6%] vs 22/527 [4.2%]; OR, 2.16; 95% CI, 0.93-5.01; adjusted P = .109). Granular cause-of-failure variables were available for 77 of 93 granted decisions (82.8%) but for only 13 of 527 denied decisions (2.5%); given this imbalance, they were summarized descriptively. Among granted decisions with granular coding, the most frequent patterns were "should have been operated or treated earlier" and "wrong treatment technique or method" (14 decisions each), followed by "inadequate monitoring or supervision" (10 decisions) and "incomplete or not initiated treatment or investigation" (9 decisions). Administrative decision grounds and failure-process domains are summarized in Fig. 3. Among denied decisions, the leading grounds were no failure in treatment (346/527, 65.7%) and no causal link (159/527, 30.2%); no failure in diagnosis accounted for 16 of 527 denied decisions (3.0%). Among granted decisions, 70 of 93 (75.3%) were attributed to failure in treatment, 7 of 93 (7.5%) to failure in diagnosis, 5 of 93 (5.4%) to the statutory exception clause, and 4 of 93 (4.3%) to infection. These are administrative decision grounds and are distinct from the clinically oriented failure-process domains. A broader failure-process domain could be assigned from coded variables and narrative review in 83 of 93 granted decisions; 10 remained unclassifiable. The most frequent failure-process domains among granted decisions were preoperative or diagnostic (35/93, 37.6%), intraoperative or procedural (22/93, 23.7%), and perioperative or postoperative management (13/93, 14.0%). Smaller categories included follow-up or surveillance (4/93, 4.3%), system-level, communication, or competence-related failures (5/93, 5.4%), and other or insufficiently specified decisions (4/93, 4.3%).

**Fig. 3:**
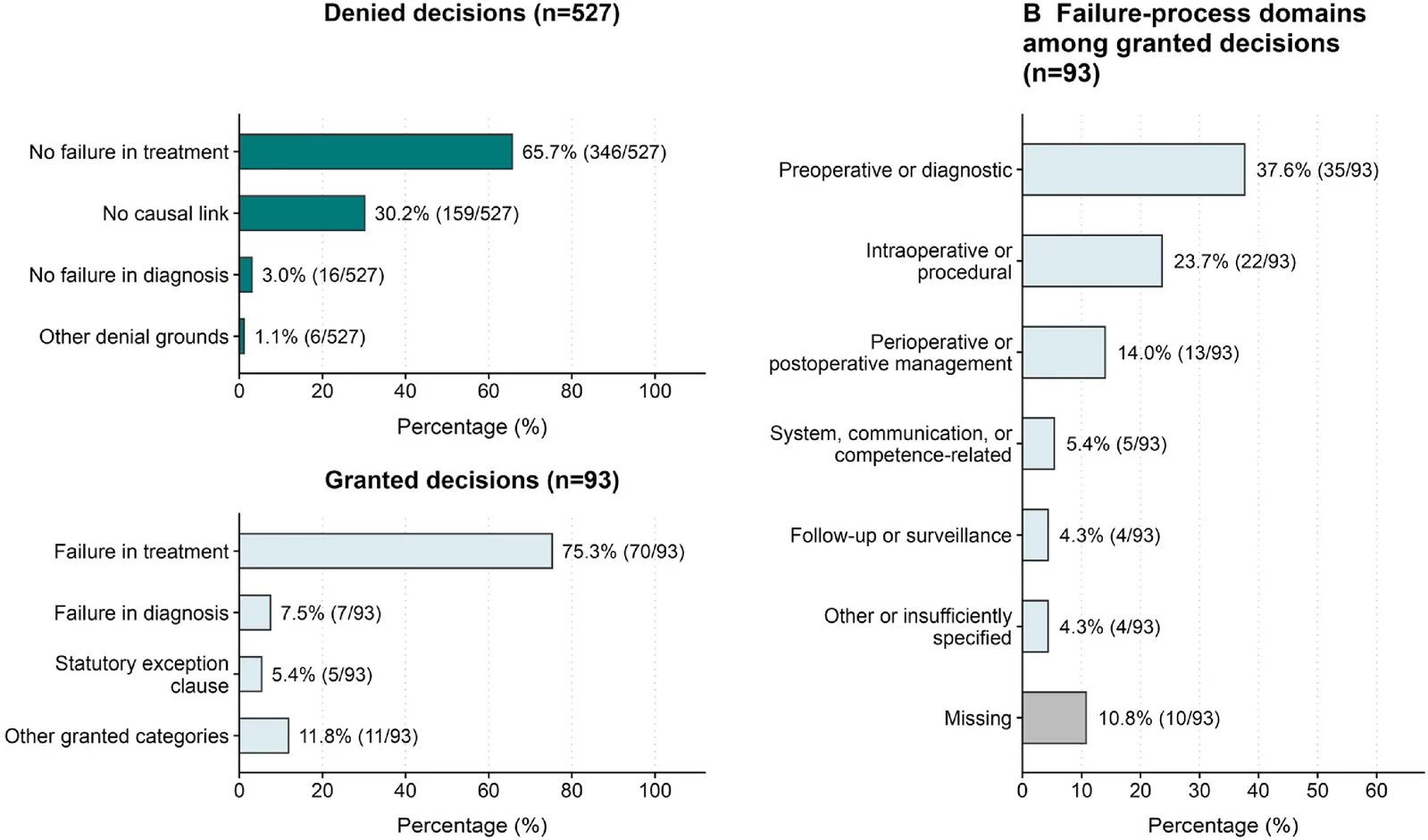
Administrative decision grounds and failure-process domains among compensation decisions. Panel A shows the administrative NPE decision grounds for denied and granted decisions. Panel B shows the broader clinically oriented failure-process domains among granted decisions, derived from coded variables and narrative review. The two panels therefore represent different levels of classification. Failure-process classification could be assigned in 83 of 93 granted decisions. The 10 cases shown as “Missing” in Panel B represent decisions in which a failure-process domain could not be classified.

### 3.4 Time-sensitive conditions

In the restricted comparison among decisions with assignable emergency status, emergency presentations included trauma, nontraumatic subarachnoid or intracerebral hemorrhage, and acute ischemic stroke interventions identified by intracranial reperfusion and/or decompressive cranial surgery. Compensation was granted in 22.4% of emergency presentations and 14.2% of nonemergency comparator presentations (OR, 1.74; 95% CI, 0.90-3.37; Fisher exact P = .12).

### 3.5 Time trends and compensation amounts

Temporal and economic analyses were based on the separate aggregated 503-decision dataset (Table 2). Grant rates were similar in 2015-2019 and 2020-2024 (17.1% vs 14.7%; OR, 1.20; 95% CI, 0.74-1.93; P = .537), with no evidence of year-to-year heterogeneity across 2015-2024 (P = .443). Among 74 fully paid compensated decisions, total compensation amounted to NOK 124 million, with a mean payout of NOK 1.7 million and a median payout of NOK 498,000.

**Table 2:** Temporal trends and compensation amounts in the aggregated dataset.

Measure	Value
Aggregated decisions, n	503
Grant rate, 2015–2019	17.1%
Grant rate, 2020–2024	14.7%
Period comparison	P = .537
Year-to-year heterogeneity, 2015–2024	P = .443
Fully paid compensated decisions, n	74
Total compensation	NOK 124 million, €11.4 million
Mean payout	NOK 1.7 million, €156,000
Median payout	NOK 498,000, €45,600

**Note.** Temporal and economic analyses were based on a separate aggregated dataset of 503 decisions and could not be linked at the individual-decision level to the main 620-decision cohort. Euro equivalents are approximate.

## 4 Discussion

### 4.1 Main findings

In this nationwide 10-year analysis of 620 neurosurgery-related compensation decisions from NPE, compensation was granted in 15.0% of decisions. Grant rates differed across neurosurgical domains, with higher proportions in cerebrospinal fluid and shunt disorders and, descriptively, trauma than in nontraumatic spinal disorders, tumors, and cerebrovascular disorders. Nontraumatic spinal disorders generated the largest volume of decisions but had a low grant rate; within this domain, cervical degenerative disease showed particularly low compensability. This discrepancy between complaint volume and compensability is important: a high number of complaints does not necessarily indicate a similarly high burden of compensable failure. Among granted decisions, failure-process mapping showed that preoperative or diagnostic failures were most frequent, followed by intraoperative or procedural failures and perioperative or postoperative management failures. These findings support a central distinction: compensation decisions should not be interpreted as neurosurgical complication rates. NPE decisions identify a narrower subset of events in which injury was judged avoidable, causally linked to care, and compensable within a defined administrative framework. Poor outcomes may reflect disease severity, recognized procedural risk, or uncertain causation and therefore may not lead to compensation. Conversely, granted decisions may arise from delayed diagnosis, inadequate investigation, insufficient monitoring, inappropriate treatment decisions, or failures in follow-up and communication rather than from isolated technical operative error alone. Administrative decision grounds and the clinically oriented failure-process domains represent different levels of classification; in particular, the administrative category of treatment failure is broader than technical operative error. Among denied decisions, no causal link accounted for 159 of 527 decisions (30.2%), indicating that causation represents a major filtering step in the compensation assessment. An adverse outcome or an alleged deficiency in care is insufficient for compensation when a causal relationship with the resulting injury cannot be established. Process-of-care problems were the most frequent claim-level event category overall but were proportionally more common among denied than granted decisions, suggesting that perceived problems in care were insufficient unless linked to a compensable failure and causal relationship. Neurologic injury codes were underrepresented among granted decisions, whereas system-, medication-, or patient-information-related events were overrepresented. Compensation was therefore not driven simply by neurological harm or dissatisfaction with care, but by adjudicated avoidability, causation, and compensability.

### 4.2 Clinical domains and compensable failure patterns

The domain-specific findings suggest that NPE data primarily signal vulnerability along the care pathway rather than procedural risk alone. In cerebrospinal fluid and shunt disorders, outcomes often depend on timely recognition of shunt malfunction or evolving hydrocephalus, appropriate imaging, reliable monitoring, and continuity of follow-up. These features may explain why this domain had the highest grant rate. Trauma may follow a similar logic because triage, imaging, escalation, and operative decision-making often occur within narrow time windows and along relatively explicit care pathways and guideline-based expectations. By contrast, degenerative spine disease generated many decisions but comparatively few awards, likely reflecting high clinical volume, chronic symptom burden, expectation-sensitive outcomes, and greater uncertainty in causal attribution. This was particularly evident for cervical degenerative disease, which accounted for most nontraumatic spinal decisions but had a substantially lower grant rate than the remaining spinal diagnoses. Taken together, these patterns indicate that compensability in neurosurgery is not determined simply by clinical severity, technical complexity, or the presence of neurological injury. Rather, compensation depends on whether the diagnostic pathway, treatment timing, monitoring, follow-up, communication, or treatment choice can be judged to have fallen below the compensable standard within the no-fault framework.

### 4.3 Compensation pathway families and medico-legal signals

The Norwegian findings should be interpreted within the compensation pathway family that produced them. Medico-legal signals are not generated by clinical events alone, but by the institutional pathway through which harm is submitted, reviewed, adjudicated, and translated into compensation. Norway belongs to a Scandinavian no-fault pathway family, together with related systems in Sweden and Denmark, in which compensation is handled administratively rather than through court-based negligence litigation (In English, Pukk-Härenstam et al., 2008, Ministry of Health and Care Service, 2007, Tilma et al., 2015, Tilma et al., 2016). These systems share reduced emphasis on proving individual negligence and a broader orientation toward system learning. They differ from hybrid administrative pathways such as those in France and Belgium (Barbot et al., 2014, Faire une demande à notre, Procédure d), from predominantly fault-based European models such as Italy and Germany (Candido et al., 2023, Stauch et al., 2013), and from litigation-backed or tort-based systems such as England and the United States (Bal, 2009, NHS Resolution annual report and). These differences are not merely procedural: they shape which injuries are submitted, how harm is framed, what evidence is required, and which failures become visible as compensable. Raw claim counts or compensation rates should therefore not be compared across countries without accounting for pathway architecture, access thresholds, institutional legitimacy, and professional culture. Survey-based indicators cannot establish causality, but variation in public trust, perceived health care quality, and neurosurgical ethico-legal practice suggests that similar clinical episodes may generate different medico-legal signals across systems (Eurobarometer. Accessed May 25, European Social Survey European Research, 2026, Mathiesen et al., 2022, OECD, 2024, Trust in public institutions, 2024). The Norwegian data should therefore be read not as a universal measure of neurosurgical risk, but as the output of a specific national no-fault framework. No-fault systems may offer particular value for patient-safety learning because they are less centered on individual blame and may make pathway-level failures more visible. Scandinavian studies suggest both the promise and limits of this approach: compensation data may help identify preventable injury when linked to hospital volumes or registry denominators, but they do not capture the same information as clinical registries and should not be treated as stand-alone mirrors of quality(Öhrn et al., 2011, Pukk-Härenstam et al., 2008, Smeby et al., 2015, Tilma et al., 2015, Tilma et al., 2016). Compensation data can contribute to safety intelligence when integrated with clinical registries, procedural denominators, reporting systems, and governance structures. Whether different medico-legal climates also influence neurosurgical training, referral behavior, documentation burden, or willingness to undertake high-risk procedures remains hypothesis-generating.

### 4.4 Limitations

First, the dataset lacked denominators for total neurosurgical activity and cannot estimate incidence per operation, admission, or care pathway. A procedure-based denominator would also not be directly comparable with the full cohort because claims may arise from diagnostic assessment, nonoperative management, surveillance, or follow-up. Second, it included only events that entered the compensation pathway and cannot capture potentially compensable injuries for which no claim was submitted or completed. Third, temporal and payment analyses relied on a separate aggregated dataset not linkable to the main cohort at the individual-decision level. Fourth, cause-of-failure coding was more complete among granted than denied decisions, reflecting administrative processing rather than true differences in mechanisms. More broadly, compensation-decision data capture only failures visible within the compensation process, and prior Norwegian work suggests that compensation-eligible adverse events are not always consistently incident-reported or documented in the medical record9. Finally, international comparisons are interpretive, not causal.

## 5 Conclusion

In this nationwide neurosurgical series from a no-fault compensation system, compensation was granted in 15.0% of decisions and was driven primarily by avoidable failures across the care pathway rather than adverse outcomes alone. Clinically oriented failure-process mapping showed that compensable failures occurred across multiple stages of the care pathway and were not limited to technical operative events. Compensation decision data should therefore not be interpreted as surgical complication rates, but as system-filtered signals of compensable and avoidable harm shaped by adjudicatory framework and system design.

## Ethics declaration

This study was performed in compliance with relevant laws, regulatory frameworks and guidelines where the research took place. Ethics committee approval was not required under relevant laws and institutional guidelines. This study used anonymized administrative data provided by NPE following formal approval. Because no identifiable patient data were used, ethical committee review and informed consent were not required under applicable regulations.

## Ethics approval and informed consent

This study used anonymized administrative data provided by NPE following formal approval. Because no identifiable patient data were used, ethical committee review and informed consent were not required under applicable regulations.

## Data availability

The data underlying this study were obtained from the Norwegian System of Patient Injury Compensation following formal approval and are not publicly available because they consist of administrative patient-injury compensation records. Aggregated or derived data may be made available from the corresponding author upon reasonable request, subject to permission from the data provider.

## Funding and disclosures

This research received no specific grant from any funding agency in the public, commercial, or not-for-profit sectors. The authors report no conflict of interest concerning the materials or methods used in this study or the findings specified in this paper.

## Declaration of competing interest

The authors declare that they have no known competing financial interests or personal relationships that could have appeared to influence the work reported in this paper.
