# Supplementary material for: Beyond complication rates: Nationwide neurosurgical compensation claims in a No-fault system
Source: Brain Spine. 2026 Oct 7;6:107470. doi: 10.1016/j.bas.2026.107470 (PMC13683439; doi:10.1016/j.bas.2026.107470)
Supplement: Supplementary file 1 [file mmc1.pdf]

STROBE Statement—Checklist of items that should be included in reports of observational studies

	Item No.	Recommendation	Page No.	Relevant text from manuscript
Title and abstract	1	(a) Indicate the study's design with a commonly used term in the title or the abstract	1	We conducted a retrospective observational study of neurosurgery-related compensation decisions issued in Norway between 2015 and 2024.
		(b) Provide in the abstract an informative and balanced summary of what was done and what was found	1	Covered in the conclusion of the abstract.
Introduction				
Background/rationale	2	Explain the scientific background and rationale for the investigation being reported	2 & 3	Covered in "Introduction" Lines 47-62.
Objectives	3	State specific objectives, including any prespecified hypotheses	2 & 3	Covered in "Introduction" Lines 58-62
Methods				
Study design	4	Present key elements of study design early in the paper	3	Covered under "Study Design, Reporting, and Data Source" Lines 64-85.
Setting	5	Describe the setting, locations, and relevant dates, including periods of recruitment, exposure, follow-up, and data collection	3	Same as above. Lines 64-85.
Participants	6	(a) Cohort study—Give the eligibility criteria, and the sources and methods of selection of participants. Describe methods of follow-up<br>Case-control study—Give the eligibility criteria, and the sources and methods of case ascertainment and control selection. Give the rationale for the choice of cases and controls<br>Cross-sectional study—Give the eligibility criteria, and the sources and methods of selection of participants	3-5	Covered well in the entirety of "Methods" Lines 63-140.

		(b) Cohort study—For matched studies, give matching criteria and number of exposed and unexposed<br>Case-control study—For matched studies, give matching criteria and the number of controls per case		Not relevant, our paper is not a matched cohort or case-control.
Variables	7	Clearly define all outcomes, exposures, predictors, potential confounders, and effect modifiers. Give diagnostic criteria, if applicable	3-5	Covered well in the entirety of "Methods"<br>Lines 63-140.
Data sources/ measurement	8*	For each variable of interest, give sources of data and details of methods of assessment (measurement). Describe comparability of assessment methods if there is more than one group	3	Covered under "Study Design, Reporting, and Data Source".<br>Lines 67-68
Bias	9	Describe any efforts to address potential sources of bias	11	Covered under "Limitation"<br>Lines 300-313
Study size	10	Explain how the study size was arrived at	3	Covered under "Study Design, Reporting, and Data Source".<br>Lines 81-82.

Continued on next page

Quantitative variables	11	Explain how quantitative variables were handled in the analyses. If applicable, describe which groupings were chosen and why	4	Covered under "Variables and classification" Lines 86-113
Statistical methods	12	(a) Describe all statistical methods, including those used to control for confounding	5	Covered under "statistical analysis" Lines 140-148
		(b) Describe any methods used to examine subgroups and interactions	5	Same as above.
		(c) Explain how missing data were addressed	3	Missed data was not explicitly stated. No imputation of missing data was performed; analyses were based on available data provided by NPE.
		(d) Cohort study—If applicable, explain how loss to follow-up was addressed<br>Case-control study—If applicable, explain how matching of cases and controls was addressed<br>Cross-sectional study—If applicable, describe analytical methods taking account of sampling strategy		Not relevant. There was no longitudinal follow up or matched sampling strategy.
		(e) Describe any sensitivity analyses		Not relevant. No formal sensitivity analysis is reported in the manuscript.
Results				
Participants	13*	(a) Report numbers of individuals at each stage of study—eg numbers potentially eligible, examined for eligibility, confirmed eligible, included in the study, completing follow-up, and analysed	5	The final analytic cohort comprised 620 compensation decisions, including 93 granted decisions (15.0%) and 527 denied decisions (85.0%)
		(b) Give reasons for non-participation at each stage		Not relevant, dataset consisted of the eligible NPE descision available for our specified time period.
		(c) Consider use of a flow diagram		Figure 1 presente the study cohort and case distribution.
Descriptive data	14*	(a) Give characteristics of study participants (eg demographic, clinical, social) and information on exposures and potential confounders	5	Covered under "Results" Line 141-156.

		(b) Indicate number of participants with missing data for each variable of interest		There were no missing data for the main variables of interest in the NPE database provided.
		(c) Cohort study—Summarise follow-up time (eg, average and total amount)		Not applicable. Our paper is not a case-control study.
Outcome data	15*	Cohort study—Report numbers of outcome events or summary measures over time	6-8	Covered under "Results" Lines 141-208
		Case-control study—Report numbers in each exposure category, or summary measures of exposure		Not applicable. This was not a case control study
		Cross-sectional study—Report numbers of outcome events or summary measures		Not applicable. This was not a cross sectional participant survey.
Main results	16	(a) Give unadjusted estimates and, if applicable, confounder-adjusted estimates and their precision (eg, 95% confidence interval). Make clear which confounders were adjusted for and why they were included	5-6	Covered in "Results" Lines 141-156
		(b) Report category boundaries when continuous variables were categorized	3-4	Covered in figure 1, and under variables and classifications. Lines 86-113
		(c) If relevant, consider translating estimates of relative risk into absolute risk for a meaningful time period		Not relevant. Relative risk estimates requiring translation into absolute risk were not the principal measures reported.

Continued on next page

Other analyses	17	Report other analyses done—eg analyses of subgroups and interactions, and sensitivity analyses	3-4	Covered in figure 1 and 2, and under variables and classifications. Lines 86-113
**Discussion**				
Key results	18	Summarise key results with reference to study objectives	8-9	Summarized under "Discussion" Lines 221-253
Limitations	19	Discuss limitations of the study, taking into account sources of potential bias or imprecision. Discuss both direction and magnitude of any potential bias	10-11	Bias and limitation is covered under "Limitation" Lines 300-314
Interpretation	20	Give a cautious overall interpretation of results considering objectives, limitations, multiplicity of analyses, results from similar studies, and other relevant evidence	8-11	Covered in the entirety of discussion Lines 221-313
Generalisability	21	Discuss the generalisability (external validity) of the study results	10-11	Covered under "limitation" Lines 311-313.
**Other information**				
Funding	22	Give the source of funding and the role of the funders for the present study and, if applicable, for the original study on which the present article is based	11	No external funding was received for this study.

*Give information separately for cases and controls in case-control studies and, if applicable, for exposed and unexposed groups in cohort and cross-sectional studies.

Note: An Explanation and Elaboration article discusses each checklist item and gives methodological background and published examples of transparent reporting. The STROBE checklist is best used in conjunction with this article (freely available on the Web sites of PLoS Medicine at http://www.plosmedicine.org/, Annals of Internal Medicine at http://www.annals.org/, and Epidemiology at http://www.epidem.com/). Information on the STROBE Initiative is available at www.strobe-statement.org.
